# Supplementary figures and images for: Prognostic stratification in hepatocellular carcinoma using a telomerase-related lncRNA signature derived from TCGA database
Source: PLoS One. 2026 Mar 6;21(3):e0339415. doi: 10.1371/journal.pone.0339415 (PMC12965591; doi:10.1371/journal.pone.0339415)

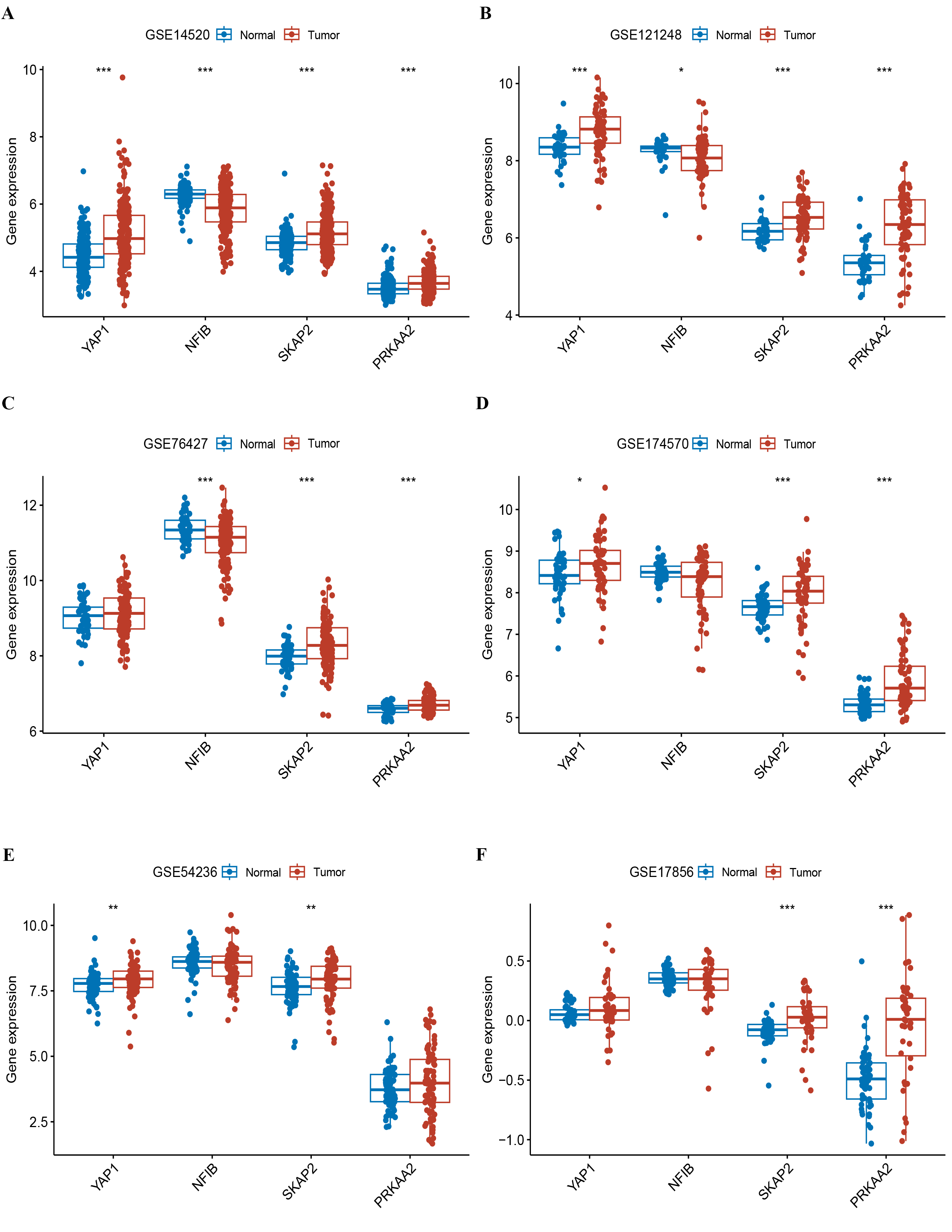

Supplement: S8 Fig — (A) GSE14520 dataset. (B) GSE121248 dataset. (C) GSE76427 dataset. (D) GSE174570 dataset. (E) GSE54236 dataset. (F) GSE17856 dataset. (TIF) [file pone.0339415.s008.tif]
