# Supplementary material for: Prognostic stratification in hepatocellular carcinoma using a telomerase-related lncRNA signature derived from TCGA database
Source: PLoS One. 2026 Mar 6;21(3):e0339415. doi: 10.1371/journal.pone.0339415 (PMC12965591; doi:10.1371/journal.pone.0339415)
Supplement: Attachment — Submitted filename: Plos ONE TRLs.docx [file pone.0339415.s018.docx]

**Peer Review Comments**

**Overview:**

This manuscript presents a four-lncRNA telomerase-related signature (AC026356.1, AL512598.1, CYTOR, and MIR210HG) for prognostic stratification in hepatocellular carcinoma (HCC), using TCGA data and limited RT-qPCR validation (n = 15). The concept of telomerase-related lncRNAs (TRLs) is relevant and aligns with current interests in molecular oncology and bioinformatics-driven biomarker research. However, despite its technical rigor, the methodological presentation should be simplified to enhance readability and comprehension. The clinical interpretation needs to be discussed in greater depth, with stronger correlation to real-world clinical application and past references. The manuscript structure also requires revision in accordance with author guidelines to improve clarity and facilitate publication. Furthermore, as the study is retrospective and includes only a small sample number (15 cases), the authors should avoid overestimating their conclusions. The title should reflect this limitation, for example, by indicating it as a *pilot* or *initial study*. **Minor Revision**

**Structure, Language, and Formatting**

- The manuscript does not fully follow *PLOS ONE* author guidelines for structure and formatting.
- Language is generally understandable but verbose. Sentences exceeding 35–40 words reduce clarity and scientific flow. Moderate English editing is strongly recommended.
- Please maintain consistent capitalization and abbreviation style (e.g., “telomerase-related lncRNAs (TRLs)” used consistently throughout).

**Novelty and Relevance**

- The research question, linking TRLs to prognosis and immune response in HCC, is timely and relevant. However, its novelty is incremental, not conceptual.
  - Similar lncRNA-based prognostic models (ferroptosis-, m6A-, or immune-related) have been reported; authors should clarify how this TRL model adds new mechanistic or clinical insight.
- Given the limited validation (only 15 patient samples), the title and conclusions should be tempered. I recommend revising the title to include “a pilot study” or “an initial exploratory analysis” to accurately represent the scale of the dataset.

**Methodological**

- Data and Analysis Pipeline
  - The process for identifying the 4-TRL signature involves multiple regression and filtering steps (WGCNA, LASSO, Cox). However, the number of lncRNAs filtered at each step is inconsistent (24 → 175 → 11 → 4). Please clarify this sequence in a detailed schematic or supplementary table.
  - Normalization and batch correction methods (e.g., FPKM vs. TPM, ComBat) are not explicitly described, especially when integrating TCGA and GEO datasets.
  - Please provide exact R package versions, thresholds, and randomization seeds used for training/testing partitioning to ensure reproducibility.
- Experimental Cohort: The RT-qPCR validation with 15 paired samples is underpowered for inferential conclusions. The authors should:
  - Rephrase results to indicate exploratory verification rather than definitive validation.
  - Clearly state this limitation in the abstract, discussion, and conclusion.
  - Avoid overgeneralizing clinical applicability.
- Functional Validation: The mechanistic claim that *AC026356.1 promotes hepatocarcinogenesis via the miR-126-5p/YAP1 axis* is based solely on bioinformatic prediction. Experimental evidence (e.g., siRNA knockdown, luciferase reporter assays) is absent.
  - The authors should acknowledge this explicitly and propose it as a future direction for a prospective study rather than a confirmed pathway.

**Discussion and Interpretation**

- Overinterpretation: Statements such as *“the 4-TRLs signature could inform personalized therapy”* are premature and unsupported by data. Replace with a more cautious phrasing, e.g., *“may provide a potential framework for future prognostic modeling.”*
- Missing Context and Comparison
  - Compare the model’s predictive accuracy to published lncRNA signatures in HCC (ferroptosis-, m6A-, or pyroptosis-related).
  - Discuss potential biological links between telomerase activity and immune modulation in HCC (e.g., Treg recruitment, NK dysfunction).
  - Elaborate on how telomerase-associated pathways intersect with immune checkpoints (CTLA-4, PD-1, LAG-3) observed in this study.
- Limitations, Please expand the limitations section to include:
  - Lack of independent or prospective cohort validation.
  - Small sample size (n = 15) for qPCR confirmation.
  - Potential population bias from the TCGA (predominantly Western, non-viral HCC).
- Structure and Clarity: The discussion would benefit from clearer subheadings:
  - Clinical relevance and comparison to existing models.
  - Potential mechanisms linking TRLs and immune response
  - Therapeutic implications and limitations

**Additional Recommendations**

- Manuscript Structure: Please adhere strictly to *PLOS ONE* formatting (Abstract, Introduction, Materials and Methods, Results, Discussion, Acknowledgments, References).
- AI Usage Disclosure: If generative AI tools assisted in writing or editing, please acknowledge this in the “Acknowledgment” or “Methods – Writing Assistance” section, as per PLOS policy on AI transparency.
