# Supplementary material for: Prognostic stratification in hepatocellular carcinoma using a telomerase-related lncRNA signature derived from TCGA database
Source: PLoS One. 2026 Mar 6;21(3):e0339415. doi: 10.1371/journal.pone.0339415 (PMC12965591; doi:10.1371/journal.pone.0339415)
Supplement: Attachment — Submitted filename: Response to Reviewers.docx [file pone.0339415.s020.docx]

Dear Editors and Reviewers:

Thank you for your letter and for the viewers' comments concerning our manuscript entitled "Prognostic stratification in hepatocellular carcinoma using a telomerase-related lncRNA signature derived from TCGA database" (Reference: PONE-D-25-52401). Those comments are all valuable and very helpful for revising and improving our paper, as well as the important guiding significance to our research. We have studied comments carefully and have made corrections which we hope will meet with approval. The main corrections in the paper and the responses to reviewer's comments are as follows:

**Academic editor**

*1. Please ensure that your manuscript meets PLOS ONE's style requirements, including those for file naming. The PLOS ONE style templates can be found at https://journals.plos.org/plosone/s/file?id=wjVg/PLOSOne_formatting_sample_main_body.pdf and [https://journals.plos.org/plosone/s/file?id=ba62/PLOSOne_formatting_sample_title_authors_affiliations.pdf](https://track.editorialmanager.com/CL0/https://journals.plos.org/plosone/s/file?id=ba62/PLOSOne_formatting_sample_title_authors_affiliations.pdf/1/010f019a810afd4b-6be24daf-79d4-4016-a8ab-236e9e1d53d4-000000/7y4Le8SIyUimxkas9pCx1T1vItr_BtuyI1RlDSb4dHI=237)*

**Response:** We have carefully revised the manuscript to ensure it fully complies with PLOS ONE's style requirements, including the file naming conventions.

*2. Please note that PLOS One has specific guidelines on code sharing for submissions in which author-generated code underpins the findings in the manuscript. In these cases, we expect all author-generated code to be made available without restrictions upon publication of the work. Please review our guidelines at https://journals.plos.org/plosone/s/materials-and-software-sharing#loc-sharing-code and ensure that your code is shared in a way that follows best practice and facilitates reproducibility and reuse.*

**Response:** In accordance with PLOS ONE's policy on code sharing, we have deposited the custom R code underpinning the bioinformatic analyses in a public repository (Zenodo) and provided the access link in the Data Availability Statement to ensure full reproducibility.

*3.Please amend the manuscript submission data (via Edit Submission) to include author Jianlie Yuan.*
**Response:** We have amended the manuscript submission data in the online system to include the author under the correct spelling: Jianlei Yuan.

*4.Please amend your authorship list in your manuscript file to include author Jianlei Yuan.*
**Response:** We have amended the authorship list in our manuscript file to the correct spelling: Jianlei Yuan.

*5. Please upload a new copy of Figures 1, 2, 3, 8, 10, and 11 as the details are not clear. Please follow the link for more information:  <https://journals.plos.org/plosone/s/figures>*

**Response:** We have re-exported and uploaded new, high-resolution versions of Figures 1, 2, 3, 8, 10, and 11 as per the journal's figure guidelines to ensure all details are clear.

We believe that we have fully addressed all concerns raised. The revisions have greatly improved the clarity, rigor, and overall quality of our work. Thank you again for the opportunity to revise our manuscript for consideration in PLOS ONE.

**Reviewer 1**

*This study centers on hepatocellular carcinoma (HCC) and systematically constructs a prognostic model based on four telomerase-related long non-coding RNAs (TRLs). It comprehensively investigates their predictive value in prognosis stratification, immune infiltration, tumor mutation burden (TMB), and drug sensitivity, supported by preliminary RT-qPCR validation. The manuscript is structurally sound, the analytical workflow is clearly presented, and the topic is innovative. The proposed model shows clinical promise; however, revisions are necessary in areas including data source utilization, experimental design, terminology consistency, and mechanistic interpretation.*

*Major comments
1.The current model is developed and validated entirely based on TCGA internal data, which poses a risk of overfitting. I suggest incorporating independent external datasets, such as those from ICGC or GEO, to enhance the model’s robustness and generalizability.*

**Response:** We sincerely thank the reviewer for raising this crucial point regarding the general applicability of our predictive model. We fully acknowledge the importance of external validation. However, most publicly available expression matrices lack standardized lncRNA annotation files and often do not include specific lncRNA probes, which prevented us from directly validating the 4-TRLs signature in independent external datasets by obtaining expression matrices for these four TRLs. To proactively address this limitation, we designed an alternative validation strategy using available datasets. Within the proposed lncRNA-miRNA-mRNA regulatory axis, the four TRLs function upstream of key TRGs. We reasoned that validating these downstream TRGs in external cohorts could provide indirect support for the biological relevance of our overall model. Accordingly, we systematically analysed six independent GEO datasets (GSE14520, GSE121248, GSE76427, GSE174570, GSE54236, and GSE17856) to examine the expression patterns of key TRGs within our predicted lncRNA-miRNA-mRNA network (Lines 106-110; 671-673). As shown in S8 Fig, these TRGs demonstrated consistent differential expression across multiple independent cohorts, providing indirect validation for our 4-TRLs signature model. While we acknowledge that this approach does not replace direct validation of the lncRNA signature, we believe that this robust, multi-cohort confirmation of key downstream elements in our model substantially strengthens its generalizability and alleviates concerns regarding overfitting. We thank the reviewer again for their valuable insights.

*2.The LASSO regression procedure for feature selection would benefit from greater transparency. The manuscript should clarify whether the optimal lambda value was chosen based on the minimum cross-validation error (min criterion) or the one-standard-error rule (1-SE criterion). This information is critical for evaluating the model's balance between simplicity and predictive accuracy.*

**Response:** We thank the reviewer for their valuable comments regarding the selection of the lambda value in LASSO regression for feature selection. In our study, the optimal lambda value was determined through cross-validation using the cv.glmnet function in R. As shown in our code, the specific implementation was as follows:

cvfit <- cv.glmnet(x, y, family = "cox", maxit = 1000)

coef <- coef(fit, s = cvfit$lambda.min)

The selection was based specifically on the minimum cross-validation error criterion (min criterion), corresponding to cvfit$lambda.min, which identifies the lambda value that minimizes the cross-validation error. Although the one-standard-error rule (1-SE rule, cvfit$lambda.1se) was also visualized in the accompanying figure for completeness, the final model was constructed using lambda.min to ensure optimal predictive accuracy. Therefore, the optimal lambda value in our analysis was selected based on the minimum cross-validation error criterion. This methodological detail has been explicitly stated in the Materials and methods section of our manuscript (Lines 165-167). We appreciate the reviewer's insightful suggestion, which has enabled us to improve the clarity and transparency of our methodology description.

*3.The SHAP analysis provides intriguing insights into feature importance within the prognostic model. However, the clinical relevance of these findings remains somewhat abstract. A clear explanation should be added regarding how SHAP-derived feature contributions could translate into practical clinical decision-making or patient stratification strategies in hepatocellular carcinoma management.*

**Response:** We thank the reviewer for this insightful comment regarding the clinical relevance of SHAP analysis in our prognostic model. We have now added the following discussion section (Lines 735-746) to address how SHAP-derived feature contributions could translate into clinical practice for HCC management: "Subsequently, we applied SHAP analysis to the 4-TRLs signature to enhance its interpretability and potential clinical applicability. In our analysis, top-ranking features identified by SHAP, such as MIR210HG and CYTOR, demonstrated substantial influence on prognostic outcomes. Features with consistently positive SHAP values among high-risk patients indicate potential drivers of unfavourable prognosis. This information holds clinical relevance in several aspects: first, for patient stratification, clinicians could prioritise close monitoring and early intervention for patients exhibiting elevated levels of high-risk features with strongly positive SHAP contributions. Second, the directionality of SHAP values helps elucidate which molecular features may be driving individual patient outcomes, potentially informing targeted therapeutic strategies." We sincerely appreciate this valuable suggestion, which has significantly strengthened the clinical translation aspect of our study.

*4. It is suggested to compare the current telomerase-based lncRNA model with other published prognostic models based on m6A-, ferroptosis-, or cuproptosis-related lncRNAs. Such comparisons would help emphasize the novelty and potential advantages of the proposed model.*

**Response:** We sincerely appreciate the reviewer's valuable suggestion regarding the comparative analysis of our 4-TRLs model with other prognostic signatures. As shown in S2 Fig, we compared our 4-TRLs signature with 16 published models (including m6A-, ferroptosis-, and cuproptosis-related signatures) in the TCGA cohort. The results revealed that our 4-TRLs signature achieved superior predictive performance for 3-year (AUC = 0.759) and 5-year (AUC = 0.770) survival, outperforming all other signatures, while maintaining competitive accuracy for 1-year prediction (AUC = 0.744) (Lines 385-392). Furthermore, detailed information for each of the 16 published models—including PMID, figure panel location, biological function, model abbreviation, and AUC values at 1-, 3-, and 5-years—is systematically compiled in S7 Table. We thank the reviewer for this insightful suggestion, which has strengthened the novelty and translational significance of our study.

*5. The correlation analysis of clinical characteristics in the TCGA-LIHC cohort reveals "unknown" entries for key prognostic variables—including tumour grade, stage, and TNM classification—as documented in Table 1 and Figures 5B and 12K. Although the distribution of these missing values appears balanced between the training and validation sets, the methods section does not specify whether these cases were included in survival analyses or model construction, nor does it describe the strategy for handling missing data. We recommend that the authors explicitly state their approach to missing data in the "Statistical Analysis" section to enhance methodological transparency and ensure the reproducibility of the reported findings.*

**Response:** We sincerely thank the reviewer for raising this important methodological point regarding the handling of missing clinical data. We appreciate the opportunity to clarify our analytical approach, and we have now updated the "Statistical analysis" section accordingly to enhance methodological transparency (Lines 268-273). In our study, we adopted distinct strategies for handling missing clinical data in different analytical contexts, based on specific analytical objectives: For correlation analyses (presented in Table 1, Figure 5B, and Figure 12K), samples with "unknown" entries for tumour grade, stage, and TNM classification were retained to provide a comprehensive overview of the entire cohort's clinical characteristics. For prognostic model construction, we included all available HCC samples (N=370) after excluding four cases with zero survival time. Samples with "unknown" clinical features were retained in this analysis, as our LASSO-Cox model utilizes only gene expression data for risk prediction and does not require complete clinical annotation. For survival analysis in S3 Fig, we excluded samples with "unknown" clinical features when assessing the model's stratification capacity across specific clinical subgroups. This approach ensures clear interpretation of Kaplan-Meier curves by maintaining well-defined clinical categories. This stratified approach to missing data allows us to both maximize statistical power in model development and maintain analytical rigor in clinical correlation assessments. We have now explicitly detailed these procedures in the revised manuscript to ensure full reproducibility of our findings. We are grateful for this constructive suggestion, which has significantly improved the clarity and rigor of our methodological description.

*6. It is important to note that CIBERSORT relies heavily on the completeness of its reference expression signature and may introduce estimation bias when applied to highly heterogeneous tumor samples. Furthermore, relying exclusively on a single computational approach may not fully capture the complexity of the tumor immune landscape. I recommend that the authors validate these findings using additional well-established algorithms—such as EPIC, MCP-counter, or TIMER—to enhance the robustness and reliability of the immune infiltration estimates. A consensus derived from multiple methodologies would strengthen the credibility of the immunophenotypic associations reported in this study.*

**Response:** We sincerely thank the reviewer for raising this important methodological consideration regarding immune infiltration estimation. We fully agree that relying on a single computational approach may not adequately capture the complexity of the tumor immune microenvironment. In response to this valuable suggestion, we have comprehensively analyzed immune cell infiltration differences between risk groups using seven distinct, well-established algorithms — including not only CIBERSORT but also XCELL, TIMER, QUANTISEO, MCPCOUNTER, EPIC, and CIBERSORT-ABS (Lines 202-204; 523-526). As shown in the bubble plot presented in S5 Fig, the consistent patterns observed across multiple algorithms validate the reliability of our immune infiltration estimates. We have now explicitly described this multi-algorithm validation strategy in the revised Result section and included the comparative results in the S9 Table. We are grateful for this insightful suggestion, which has substantially improved the robustness and reliability of our findings.

*7. The observation that high TIDE scores correlate with the high-risk group is noted, but the biological and clinical implications are not sufficiently discussed. The authors should briefly explain how elevated TIDE scores are mechanistically linked to immunotherapy resistance, citing relevant literature to contextualize the findings within the existing immuno-oncology framework.*

**Response:** We thank the reviewer for this insightful comment. In response, we have added a dedicated paragraph in the Discussion section to elaborate on the mechanistic and clinical implications of the elevated TIDE scores in our H-R group. Specifically, we have cited recent literature to contextualize how high TIDE scores reflect key mechanisms of immunotherapy resistance, such as T-cell dysfunction and exclusion, and linked these findings to the established immuno-oncology framework in HCC (Lines 775-782). We appreciate the reviewer's valuable suggestion, which has helped strengthen our manuscript.

*8. The proposed AC026356.1–miR-126-5p–mRNA regulatory axis presented in Fig 13A-M represents a mechanistically plausible pathway yet remains computationally derived without experimental validation. I recommend that the authors explicitly acknowledge this limitation in the Discussion section, emphasizing the preliminary nature of these bioinformatic predictions and the need for functional validation to establish causal relationships.*

**Response:** We thank the reviewer for raising this important point regarding the need to acknowledge the computational nature of our proposed regulatory axis. In response to this comment, we have explicitly acknowledged this limitation in the Discussion section of our revised manuscript. Specifically, we have added the following statement: Lastly, while bioinformatic approaches have suggested that these lncRNAs work in crucial death-related pathways like cuproptosis and ferroptosis, their precise roles and molecular mechanisms in HCC remain to be verified. We also acknowledge that while the AC026356.1-miR-126-5p/YAP1 axis appear to hold at least in part a causal role, this still remains in the domain of bioinformatics. Experimental validation via siRNA knockdown, luciferase assays and rescue studies to attune whether this indeed has a functional cascade in HCC is highly crucial (Lines 848-854). We are grateful to the reviewer for this valuable suggestion, which has helped improve the transparency and rigor of our study.

*9. The manuscript describes "Internal and external validation of the 4-TRLs signature" (Line 601); however, the validation is limited to internal training/test set splits within the TCGA cohort, without inclusion of independent external cohorts—such as those from GEO databases or multi-institutional collaborations—for proper external validation. While RT-qPCR using hospital-derived samples provides valuable experimental confirmation, this constitutes technical validation rather than true external validation in an independent clinical cohort. We recommend revising the terminology to accurately reflect the validation approaches used and conducting comprehensive language polishing throughout to enhance scientific precision and readability.*

**Response:** We sincerely thank the reviewer for their insightful comments regarding the validation terminology used in our manuscript. We acknowledge that while the RT‑qPCR validation using hospital-derived samples provides valuable technical confirmation, it does not constitute true external validation in an independent clinical cohort. Accordingly, we have reframed this section to emphasize its role in pilot experimental verification rather than external validation. The subsection heading has been revised to: "Pilot experimental validation of the 4-TRL signature and internal assessment of lncRNA AC026356.1 (Lines 622-623)." In addition, we have performed comprehensive language polishing throughout the manuscript to improve scientific precision and readability. We are grateful for this constructive suggestion, which has significantly improved the accuracy and clarity of our methodological descriptions.

*10.The manuscript exhibits inconsistencies in the reporting of statistical significance, with variations in p-value formatting (e.g., "p < 0.001" versus "P < 0.001") and insufficient documentation of statistical methods in several figures. I recommend standardizing statistical reporting to the format "p < 0.001" throughout the text and explicitly specifying the statistical tests employed (e.g., log-rank test, Student's t-test) in both figure legends and the Methods section.*

**Response:** We sincerely thank the reviewer for this valuable comment regarding the standardization of statistical reporting in our manuscript. We have carefully addressed these concerns through the following revisions: We have standardized the reporting of statistical significance throughout the text by consistently using the format "p < 0.001" in all relevant sections. We have explicitly specified the statistical tests employed in all figure legends, including, for example, the log-rank test for survival analyses and Student's t-test for group comparisons (Lines 647-649; 1092-1093). We have expanded the "Statistical Analysis" subsection in the Methods section to provide a comprehensive description of all statistical methods used in the study (Lines 272-273; 283-284). We are grateful for this constructive suggestion, which has significantly improved the rigor and reproducibility of our statistical reporting.

*Minor comment
1.All acronyms should be defined at first use.*

**Response:** We sincerely appreciate the reviewer's valuable suggestion regarding acronym standardization. In response, we have carefully reviewed the manuscript to ensure all acronyms are clearly defined at first use, including: LASSO (least absolute shrinkage and selection operator), ROC (receiver operating characteristic), DEGs (differentially expressed genes), WGCNA (Weighted Gene Co-expression Network Analysis), TIDE (Tumor Immune Dysfunction and Exclusion), and IC_50_ (half-maximal inhibitory concentration). These definitions have been consistently applied throughout the text, figures, and tables. We thank the reviewer for this important suggestion, which enhances the manuscript's clarity and ensures proper interpretation of our findings.

*2.Figure titles and legends could be refined to be more concise and professionally formatted.*

**Response:** We sincerely appreciate the reviewer's valuable comments regarding the figure presentation in our manuscript. All figure titles and legends have been thoroughly revised to enhance conciseness and maintain professional formatting standards. We believe these modifications have significantly strengthened the overall quality and readability of our graphical presentations, and we are grateful for the reviewer's insightful suggestions that helped us improve the manuscript.

**Reviewer 2**

*This manuscript presents a four-lncRNA telomerase-related signature (AC026356.1, AL512598.1, CYTOR, and MIR210HG) for prognostic stratification in hepatocellular carcinoma (HCC), using TCGA data and limited RT-qPCR validation (n = 15). The concept of telomerase-related lncRNAs (TRLs) is relevant and aligns with current interests in molecular oncology and bioinformatics-driven biomarker research. However, despite its technical rigor, the methodological presentation should be simplified to enhance readability and comprehension. The clinical interpretation needs to be discussed in greater depth, with stronger correlation to real-world clinical application and past references. The manuscript structure also requires revision in accordance with author guidelines to improve clarity and facilitate publication. Furthermore, as the study is retrospective and includes only a small sample number (15 cases), the authors should avoid overestimating their conclusions. The title should reflect this limitation, for example, by indicating it as a pilot or initial study. Minor Revision*

***Structure, Language, and Formatting***

- *The manuscript does not fully follow PLOS ONE author guidelines for structure and formatting.*

**Response:** We thank the reviewer for highlighting this important issue. In response, we have thoroughly restructured the manuscript to ensure full compliance with PLOS ONE author guidelines. Key additions include the mandatory "Data Availability Statement", "Funding", and "Competing Interests" statements. The "Materials and methods" section has also been updated to include a more detailed ethics statement. We believe the manuscript now fully adheres to the journal's formatting and structural requirements.

- *Language is generally understandable but verbose. Sentences exceeding 35–40 words reduce clarity and scientific flow. Moderate English editing is strongly recommended.*

**Response:** We thank the reviewer for this constructive feedback. We have comprehensively edited the manuscript to improve conciseness. This involved breaking down overly long sentences and streamlining wording to enhance clarity and scientific flow. These revisions significantly enhance the clarity and conciseness of the scientific presentation.

- *Please maintain consistent capitalization and abbreviation style (e.g., “telomerase-related lncRNAs (TRLs)” used consistently throughout).*

**Response:** We thank the reviewer for this pertinent observation. We have conducted a systematic audit of the manuscript to ensure that all terminology, capitalization, and abbreviations strictly adhere to a consistent style. This includes the standardized presentation of "telomerase-related lncRNAs (TRLs)" and the uniform reference to our prognostic model as the "4-TRLs signature" throughout the text. We are confident that these revisions have enhanced the manuscript's clarity and consistency.

***Novelty and Relevance***

- *The research question, linking TRLs to prognosis and immune response in HCC, is timely and relevant. However, its novelty is incremental, not conceptual.*
  - *Similar lncRNA-based prognostic models (ferroptosis-, m6A-, or immune-related) have been reported; authors should clarify how this TRL model adds new mechanistic or clinical insight.*

**Response:** We thank the reviewers for their valuable insights. The novelty of this study lies in its multidimensional exploration of the mechanistic roles and clinical relevance of TRLs in HCC, distinguishing it from previous studies that primarily relied on expression profiling for prognostic modeling. Specifically, by integrating single-cell transcriptomic data, we revealed cell type-specific expression patterns of four key TRLs across distinct cellular subsets within the tumour microenvironment, offering new insights into their context-dependent functions (Lines 585-609). Furthermore, we calculated and predicted the regulatory role of lncRNA AC026356.1 in regulating downstream telomerase-related genes by adsorbing miR-126-5p, and proposed a new molecular axis of action (Lines 622-644). In addition, comparative analysis against 16 previously published HCC prognostic models demonstrated that the 4-TRLs signature achieved superior accuracy in predicting 3-year and 5-year survival, supporting its potential as a clinically applicable prognostic tool (Lines 385-392). We believe these points collectively underscore the conceptual advance of our work and are grateful for the opportunity to clarify them.

- *Given the limited validation (only 15 patient samples), the title and conclusions should be tempered. I recommend revising the title to include “a pilot study” or “an initial exploratory analysis” to accurately represent the scale of the dataset.*

**Response:** We thank the reviewer for this constructive suggestion. We agree that the sample size for experimental validation is limited. In direct response to this comment, we have revised the title to include "a pilot study" to more accurately represent the scale of our validation cohort. Furthermore, we have tempered the language in the abstract, results, and conclusion sections to avoid overstatement and to frame our findings as a promising initial validation that warrants further investigation in larger cohorts. We believe these revisions provide a more precise and balanced representation of our work. We are grateful for the reviewer's insightful comment, which has helped strengthen the manuscript (Lines 622-623).

***Methodological***

- *Data and Analysis Pipeline*
  - *The process for identifying the 4-TRL signature involves multiple regression and filtering steps (WGCNA, LASSO, Cox). However, the number of lncRNAs filtered at each step is inconsistent (24 → 175 → 11 → 4). Please clarify this sequence in a detailed schematic or supplementary table.*

**Response:** We thank the reviewer for this astute observation and the opportunity to clarify the feature selection pipeline. The reviewer is correct; there was an inconsistency in our initial description. The sequence should have been 424 → 175 → 11 → 4. To address this comment fully and enhance clarity, we have now included: A new S6 Table that provides a detailed, step-by-step breakdown of the entire filtering process, including the input, criteria, and output (Lines 364-365). We believe these additions now provide a transparent and easy-to-follow account of our model construction process.

- *Data and Analysis Pipeline*
  - *Normalization and batch correction methods (e.g., FPKM vs. TPM, ComBat) are not explicitly described, especially when integrating TCGA and GEO datasets.*

**Response:** We thank the reviewer for this important comment. We have now explicitly described the normalization methods in the revised manuscript. To address this point: TCGA Data—Both mRNA and lncRNA expression data were obtained as pre-normalized FPKM values and were log2-transformed for analysis. GEO single-cell Data—The raw UMI counts were normalized using the standard Seurat LogNormalize method (scale factor = 10,000) and scaled to z-scores. We also clarify that the TCGA and GEO datasets were analyzed separately, not integrated; hence, batch correction was not applicable. These details are now explicitly stated in the "Statistical analysis" section (Lines 274-276).

- *Data and Analysis Pipeline*
  - *Please provide exact R package versions, thresholds, and randomization seeds used for training/testing partitioning to ensure reproducibility.*

**Response:** We thank the reviewer for highlighting the need for precise documentation of the data partitioning process. We used the createDataPartition function from the caret package (version 7.0.1) for training/testing partitioning, with a random seed of 222 and a 50/50 split ratio (p = 0.5). These details have been explicitly stated in the "Materials and Methods" section of our revised manuscript to ensure complete reproducibility of the data partitioning step (Lines 158-163).

- *Experimental Cohort: The RT-qPCR validation with 15 paired samples is underpowered for inferential conclusions. The authors should:*
  - *Rephrase results to indicate exploratory verification rather than definitive validation.*
  - *Clearly state this limitation in the abstract, discussion, and conclusion.*
  - *Avoid overgeneralizing clinical applicability.*

**Response:** We extend our sincere gratitude to the reviewer for this critical and constructive comment. We fully concur that the sample size for experimental validation is limited and insufficient to draw definitive inferential conclusions. In direct response, we have thoroughly revised the manuscript to address these concerns as follows: Rephrased Interpretation—Throughout the manuscript, particularly in the Results section, we have replaced definitive terms such as "validation" and "confirmed" with more precise language, including "preliminary support" and "revealed." Explicit Statement of Limitations—We have now explicitly acknowledged the constraint of the small validation cohort in the Abstract, Discussion, and Conclusion. The Discussion includes a dedicated paragraph (Lines 838-842) emphasizing the preliminary nature of the experimental verification and the necessity for validation in larger cohorts. Avoidance of Overgeneralization—We have moderated claims regarding clinical applicability in the Abstract and Conclusion, reframing the signature as a "potential" tool and explicitly stating that its clinical utility must be confirmed through larger, prospective studies. We are confident that these revisions have significantly strengthened the manuscript by providing a more accurate, balanced, and cautious interpretation of our initial experimental findings, aligning them with the scale of the validation cohort. We are deeply appreciative of the reviewer’s insightful input, which has enhanced the quality of our work.

- *Functional Validation: The mechanistic claim that AC026356.1 promotes hepatocarcinogenesis via the miR-126-5p/YAP1 axis is based solely on bioinformatic prediction. Experimental evidence (e.g., siRNA knockdown, luciferase reporter assays) is absent.*
  - *The authors should acknowledge this explicitly and propose it as a future direction for a prospective study rather than a confirmed pathway.*

**Response:** We thank the reviewer for raising this important point regarding the need for experimental validation of the proposed lncRNA AC026356.1-miR‑126‑5p/YAP1 axis. In response to this comment, we have explicitly acknowledged this limitation in the Discussion section of our revised manuscript. Specifically, we have added the following statement: "Moreover, we acknowledge that the proposed AC026356.1-miR‑126‑5p/YAP1 axis remains computationally derived. Experimental validation through siRNA knockdown, luciferase assays, and functional rescue studies is required to establish its causal role in HCC. (Lines 851-854)." Furthermore, we have carefully revised the corresponding Results section to reframe these findings as a prospective prediction rather than an established pathway. The text now consistently uses terminology such as "predicted regulatory network" and "computational evidence" to emphasize the preliminary nature of these bioinformatic insights. We have also explicitly positioned this axis as a promising direction for future experimental investigation in hepatocellular carcinogenesis. We are grateful to the reviewer for this valuable suggestion, which has significantly improved the accuracy and transparency of our manuscript.

***Discussion and Interpretation***

- *Overinterpretation: Statements such as “the 4-TRLs signature could inform personalized therapy” are premature and unsupported by data. Replace with a more cautious phrasing, e.g., “may provide a potential framework for future prognostic modeling.”*

**Response:** We thank the reviewer for this critical correction. We agree that our original statement overinterpreted the findings and made premature claims about clinical utility. In direct response to this comment, we have replaced the overreaching conclusion throughout the manuscript, including in the abstract and discussion, with more cautious and accurate language that aligns with the current stage of our research. We believe this revised phrasing appropriately frames our findings as hypothesis-generating and emphasizes the necessity of future validation, thereby addressing the reviewer's concern about overinterpretation.

- *Missing Context and Comparison*
  - *Compare the model’s predictive accuracy to published lncRNA signatures in HCC (ferroptosis-, m6A-, or pyroptosis-related).*
  - *Discuss potential biological links between telomerase activity and immune modulation in HCC (e.g., Treg recruitment, NK dysfunction).*
  - *Elaborate on how telomerase-associated pathways intersect with immune checkpoints (CTLA-4, PD-1, LAG-3) observed in this study.*

**Response:** We thank the reviewers for their valuable comments regarding model comparison and mechanistic elaboration. We have thoroughly revised the manuscript to address these points as follows:Firstly, we have systematically compared our 4-TRLs signature against 16 published lncRNA models (Lines 385-392, S2 Fig). The details, compiled in S6 Table, demonstrate our model's competitive, and often superior, prognostic accuracy, especially for long-term survival. Secondly, we have expanded the Discussion to explore the biological links between telomerase activity and immune modulation, specifically regarding Treg recruitment and NK cell dysfunction (Lines 757-770). We discuss how telomerase-driven pathways may foster an immunosuppressive microenvironment to facilitate immune escape. Furthermore, we have incorporated a discussion on the potential mechanistic interactions between the upregulation of theimmune checkpoint (CTLA-4, LAG-3, and CD276) and telomerase activity (Lines 770-777). We believe these significant revisions have substantially strengthened the context, comparability, and mechanistic depth of our study. We are grateful for the reviewers' insightful suggestions, which have greatly improved our manuscript.

- *Limitations, Please expand the limitations section to include:*
  - *Lack of independent or prospective cohort validation.*
  - *Small sample size (n = 15) for qPCR confirmation.*
  - *Potential population bias from the TCGA (predominantly Western, non-viral HCC).*

**Response:** We thank the reviewer for raising these important methodological considerations. In response to this valuable comment, we have significantly expanded the Limitations section in our revised manuscript to specifically address these points. As now detailed in the Limitations section, we have explicitly acknowledged: The limited sample size (n=15) for our qPCR confirmation and its potential impact on the robustness of conclusions (Lines 838-842). The lack of independent or prospective cohort validation beyond the TCGA datase (Lines 842-845). Potential population biases inherent in the TCGA cohort, particularly its predominance of Western populations with non-viral HCC etiologies (Lines 845-848). We believe these revisions have strengthened the manuscript by offering a balanced perspective on both the strengths and limitations of our findings. We are grateful for this constructive suggestion, which has helped improve the scholarly rigor and transparency of our work.

- *Structure and Clarity: The discussion would benefit from clearer subheadings:*
  - *Clinical relevance and comparison to existing models.*
  - *Potential mechanisms linking TRLs and immune response*
  - *Therapeutic implications and limitations*

**Response:** We thank the reviewer for this excellent suggestion to improve the structural clarity of the Discussion. While PLOS ONE articles typically do not use formal subheadings in the Discussion, we have fully integrated the reviewer's feedback by fundamentally restructuring this section to follow the recommended logical flow. We have now introduced distinct thematic paragraphs, each beginning with a clear topic sentence, that explicitly address:

1.Through our rigorous bioinformatic screening and statistical modeling, we constructed the novel 4-TRLs signature, which was protective and exhibiting superior predictive accuracy and clinical interpretability compared with previous models (Lines 727-729).

2.The poor prognosis in the H-R group may also be a consequence of a multifactorial immunosuppressive circuitry where telomerase activity may be the conductor that orchestrates Treg recruitment, NK cell 'normalizing', and multiple immune checkpoint upregulation (Lines 751-754).

3.4-TRLs signature are associated with several pro-tumorigenic phenomena, of which proliferation, cell death as well as metabolism are established processes, and are associated functionally with telomerase, represented in example by the predicted AC026356.1/miR-126-5p axis (Lines 791-794).

We are confident that the restructured Discussion section now presents a more coherent narrative, and we are grateful for the reviewer's suggestion which has enhanced the manuscript's readability.

***Additional Recommendations***

- *Manuscript Structure: Please adhere strictly to PLOS ONE formatting (Abstract, Introduction, Materials and Methods, Results, Discussion, Acknowledgments, References).*

**Response:** We thank the reviewer for this reminder. We have now strictly adhered to the complete PLOS ONE formatting structure. In addition to the core sections (Abstract, Introduction, Materials and Methods, Results, Discussion, Acknowledgments, References) which were already in place, we have appended the required standalone statements at the end of the manuscript, including the Data availability statement, Funding, Competing interests and Author contributions. The manuscript now fully complies with the journal's formatting guidelines. We are grateful for the reviewer's vigilance in ensuring the completeness of our submission.

- *AI Usage Disclosure: If generative AI tools assisted in writing or editing, please acknowledge this in the “Acknowledgment” or “Methods – Writing Assistance” section, as per PLOS policy on AI transparency.*

**Response:** We thank the reviewer for highlighting this important policy. We have added an AI usage statement to the Acknowledgments section (Lines 874-875) clarifying that AI was used only for initial language polishing. Importantly, we conducted AI detection and professional language editing on the final manuscript, and have submitted both the AI detection report and language editing certificate as supplementary materials.

Jianlei Yuan

Cangzhou People's Hospital,

Cangzhou, Hebei,

People's Republic of China

Tel: (+86) 181 0327 6691

Email: [18103276691@163.com](mailto:18103276691@163.com)
